# Biomarkers in the early stage of PD-1 inhibitor treatment have shown superior predictive capabilities for immune-related thyroid dysfunction

**DOI:** 10.3389/fimmu.2024.1458488

**Published:** 2024-10-10

**Authors:** Jinyu Liu, Mengli Chen, Shu Li, Le Cai, Liang Ma, Qiuliang Yang, Xiaoxuan Zhang, Nan Bai, Xiaodong Wu, Zhihui Tang, Tianlin Wang

**Affiliations:** ^1^ Department of Pharmacy, Medical Supplies Center of Chinese PLA General Hospital, Beijing, China; ^2^ Department of Clinical Laboratory, The First Medical Center of Chinese PLA General Hospital, Beijing, China; ^3^ Department of Oncology, The Fifth Medical Center of PLA General Hospital, Beijing, China

**Keywords:** biomarkers, early treatment stage, cytokines, thyroid dysfunction, immunotherapy

## Abstract

**Objective:**

Hematological indicators in the early stage of PD-1 inhibitor treatment may show superior predictive ability of the occurrence of immune related adverse event (irAE) compared to the pre-treatment indicators, as the immune response is modulated during the PD-1 inhibitor treatment. The objective of this study was to investigate the predictive capabilities of biomarkers in the early treatment stage for immune related thyroid dysfunction (irTD), and explore the potential predictive cytokines.

**Methods:**

Medical records and blood test results of cancer patients treated with PD-1 inhibitor at a certain medical institution were collected. Logistic regression analysis was utilized to identify the predictive factors of irTD, ROC curves were plotted and the area under the curves (AUC) was calculated. Serum samples were collected before and during early treatment phase, cytokine detection was performed to explore potential predictive cytokines.

**Results:**

A total of 264 patients were enrolled, 58 developed irTD (21.97%), including 31 patients with thyrotoxicosis and 27 with hypothyroidism. There were no significant differences in demographic characteristics, tumor types and PD-1 inhibitors between patients with and without irTD. Multivariate logistic analysis showed that anti-thyroglobulin antibody (TgAb) (OR=2.831, 95%CI: 1.077-7.443, *P*=0.035) and anti-thyroperoxidase antibody (TPOAb) (OR=9.565, 95%CI: 3.399-26.921, *P*=0.000) in the early treatment phase were independent predictive factors for irTD, the AUC of early-stage biomarkers was larger than that of pre-treatment (0.655 vs 0.571); low level of TSH at the early stage (OR=0.162, 95%CI: 0.077-0.341, *P*=0.000) was significantly correlated with thyrotoxicosis; female (OR=3.889, 95%CI: 1.457-10.380, *P*=0.007) and positive TPOAb (OR=8.678, 95%CI: 2.656-28.357, *P*=0.000) at the early stage were significantly correlated with hypothyroidism. The AUCs of early-stage biomarkers were larger than that of pre-treatment both in thyrotoxicosis (0.812 vs 0.637) and hypothyroidism patients (0.728 vs 0.710). The increase of IL-16 (adjusted *P*=0.004), IL-12p70 (adjusted *P*=0.014), IL-17 (adjusted *P*=0.014), CCL-15 (adjusted *P*=0.014) and IL-1a (adjusted *P*=0.021) in the early treatment phase were positively correlated with irTD.

**Conclusions:**

Biomarkers at the early stage of PD-1 inhibitor treatment could predict irTD, and demonstrated stronger predictive ability compared to pre-treatment biomarkers. IL-16, IL-12p70, IL-17, CCL-15 and IL-1a could serve as potential predictive biomarkers for irTD.

## Introduction

Immune-related thyroid dysfunction (irTD) is a prevalent endocrine immune-related adverse event (irAE) induced by PD-1 inhibitors, with a higher incidence than observed in clinical trials. Patients with irTD may be asymptomatic or experience non-specific symptoms like palpitations, fatigue and weight changes, which are similar to those patients with advanced cancer. The similarity makes it challenging to differentiate the two conditions, increasing the risk of missed diagnosis (1, 2). Half of the patients develop irreversible damage at the time of irTD diagnosis, and severe cases can be life-threatening (3, 4). Therefore, predicting and identifying irTD early are crucial in the clinical management. Besides routine thyroid function monitoring, exploring potential predictive markers for irTD can effectively reduce the risk of missed diagnosis, enable early intervention, and improve quality of life.

Several studies have investigated predictive markers for irTD, but they have focused on the pre-treatment biomarkers (5, 6). Given that PD-1 inhibitors significantly impact the immune microenvironment by regulating gene and protein expression, as well as the composition of immune cell subsets, leading to changes in cellular and humoral immunity (7–9). Such modulations may precipitate in inducing irTD. Consequently, biomarkers subsequent to PD-1 inhibitor treatment are likely to offer better predictive utility when compared with pre-treatment biomarkers. Hematological markers are easily accessible and cost-effective, providing significant advantages as predictive markers. The role of cytokines in predicting irAEs has been validated (10, 11), however, researches on the predictive value of cytokines for irTDs are limited. This study aimed to investigate the predictive efficacy of hematological indicators at the early stage of PD-1 inhibitor treatment for irTD and explore the potential predictive value of serum cytokines.

## Methods

### Patients

Cancer patients who received PD-1 inhibitors at a certain medical institution from January 1, 2021 to December 31, 2023 were included. The inclusion criteria were: (1) diagnosed as tumor through histopathological examination, (2) received at least 1 cycle of PD-1 inhibitor, (3) underwent at least 2 thyroid function tests. The exclusion criteria were: (1) previously received immunotherapy, (2) patients were with hypothyroidism or thyrotoxicosis before PD-1 administration, (3) took medication of thyroid diseases simultaneously, (4) incomplete clinical characteristics.

A total of 264 patients were enrolled and divided into two groups based on occurrence of irTD: the irTD group and the non-irTD group. This study was approved by the Ethics Committee of the Chinese PLA General Hospital (S2022-184-04) and was conducted in accordance with the Declaration of Helsinki.

### Data collection

Clinical data were collected from the electronic medical record system, including gender, age, body mass index (BMI), history of thyroid disease, tumor type, type of PD-1 inhibitor, etc. Blood routine results before and at the early stage of treatment (after 1-2 cycles of treatment) were recorded, including the count of neutrophils, lymphocytes and platelets. The thyroid function indicators before and after treatment were also recorded, including free thyroxine (FT4), thyroid stimulating hormone (TSH), anti-thyroglobulin antibody (TgAb) and anti-thyroperoxidase antibody (TPOAb).

IrTDs were subcategorized according to the thyroid function test. Overt thyrotoxicosis was defined as decreased TSH below the lower reference interval with elevated FT4 above the normal reference range. Subclinical thyrotoxicosis was defined as TSH below the lower reference interval with normal FT4. Overt hypothyroidism was defined as elevated TSH above the normal reference range and decreased FT4 below the lower reference interval. Subclinical hypothyroidism was defined as elevated TSH above the normal reference range with normal FT4.

### Cytokine detection

Sera in irTD and non-irTD group before and in the early treatment stage were collected. Sera of 16 patients from each group were matched into 16 pairs at 1:1 ratio according to gender, age and tumor type and were performed cytokine detection. The human cytokine assay (QAH-CAA-2000, RayBiotech) was used to quantitatively measure the levels of 120 inflammatory and immune-related cytokines before and at early stage of treatment, and was performed strictly following the operating instructions. These cytokines mainly include inflammatory factors, chemokines, growth factors, etc., playing an important role in immunity and inflammation. These cytokines that covered the interleukin family such as IL-1, IL-17, interferons such as IFN-α, IFN-γ, chemokines such as C-C motif chemokine ligand subfamily (CCL19, CCL26), C-X-C motif chemokine ligand subfamily (CXCL9, CXCL10) and transforming growth factors such as TGF-β, were closely related to various irAEs (12). And the list of the investigated cytokines was shown in **Supplementary Material S1**.

### Statistical analysis

Continuous variables that fitted normal distribution were described as mean ± standard deviation, and differences between patients with irTDs and without irTDs were analyzed using *t*-test. Continuous variables that did not conform normal distribution were described as median and interquartile range (IQR), and differences between groups were analyzed using the Mann-Whitney U test. Categorical variables were reported as frequency and percentage, and comparisons between groups were analyzed using the chi-square test or Fisher’s exact test. Clinical characteristics and hematological indicators with *P* < 0.2 from the univariate logistic regression were included in the multivariate logistic regression to analyze the independent risk factors for irTD. ROC curves were plotted based on independent risk factors, the area under curve (AUC) was compared between pre-treatment and early-stage treatment biomarkers. A value of two-sided *P*<0.05 was indicated as statistical difference. Multiple imputation method was used for data imputation. All statistical analysis were performed using SPSS Statistics version 25 (IBM, Armonk, NY, USA) and GraphPad Prism 8 (GraphPad, San Diego, CA).

The raw data of cytokine assay were processed with background removal and normalization using Raybiotech software. The standards were averaged and normalized, the logarithmic regression equation was performed with R^2^ > 0.9 to establish the standard curve. The concentration of each samples was calculated according to the standard curve. The concentration values were analyzed using the moderated *t*-test, with the data package limma from R/Bioconductor. Differential proteins were screened using the adjusted *P* value (Benjamini-Hochberg method-corrected *P* value) and logFC (fold change), the selection criteria were: (1) logFC > log2(1.2) or < log2(0.83), with a difference threshold of 1.2 and 0.83, (2) the adj *P* value < 0.05.

## Results

### Patient characteristics

A total of 264 patients were included, 70 patients developed irAEs, and 6 patients experienced at least 2 irAEs. The irAEs included irTD (58, 21.97%), immune-related pneumonitis (7, 2.65%), myocarditis (4, 1.51%), rash (2, 0.76%), hepatitis (1, 0.38%), enteritis (1, 0.38%), hypophysitis (1, 0.38%), kidney injury (1, 0.38%) and diabetes (1, 0.38%). Most of them were grade 1-2, except 1 patient with grade 3 hepatitis and 2 patients with grade 3 rash. For the majority of grade 1-2 irAEs, the general treatment was to observe, enhance monitoring or provide symptomatic treatment. 9 patients were treated with steroids. 1 patient with myocarditis received symptomatic treatment in combination with steroids and immunosuppressive therapy. 14 patients discontinued PD-1 inhibitor due to irAEs (6 with pneumonitis, 4 with myocarditis, 2 with rash, 1 with hypophysitis, and 1 with diabetes and kidney injury), among them, 4 patients rechallenged with PD-1 inhibitor after irAE improvement. The summary of irAEs in patients treated with PD-1 inhibitor was shown in **Supplementary Table S1**.

The incidence rate of irTD was the highest among irAEs, with a median occurrence time of 63 days after the first administration of PD-1 inhibitor. Among patients, 21 had subclinical thyrotoxicosis, 10 had overt thyrotoxicosis, 9 had subclinical hypothyroidism and 18 had overt hypothyroidism. All were grade 1-2. 4 patients with hypothyroidism received L-Thyroxine, 1 with thyrotoxicosis was advised to follow a low-iodine diet and 1 received methimazole. None patients discontinued PD-1 inhibitor due to irTDs. There were no significant differences in clinical characteristics between the irTD and non-irTD group. Compared to pre-treatment, neutrophil-to-lymphocyte ratio (NLR) (*P*=0.001), platelet-to-lymphocyte ratio (PLR) (*P*=0.004) and TSH (*P*=0.000) all decreased at the early stage of treatment. There was a significant difference in pre-treatment TPOAb between two groups (*P*=0.001), and significant differences of early-stage TSH, TgAb and TPOAb were observed (*P*=0.000). The clinical characteristics were shown in **Table 1**.

**Table 1:** Patient characteristics.

Patient characteristics	All patients (n=264)	irTD group (n=58)	non-irTD group (n=206)	*P* value
**Sex (n, %)**				0.359
Male	224 (84.8)	47 (81.0)	177 (85.9)	
Female	40 (15.2)	11 (19.0)	29 (14.1)	
**Age (median, IQR)**	63 (55,70)	62 (56,69)	63 (55,70)	0.690
**BMI (kg/m^2^) (median, IQR)**	23.5 (21.2,25.8)	22.6 (21.0,24.9)	23.9 (21.4,26.0)	0.102
**History of thyroid disease (n, %)**				1.000
Yes	16 (6.1)	4 (6.9)	12 (5.8)	
No	248 (93.9)	54 (93.1)	194 (94.2)	
**Tumor type (n, %)**				0.923
Lung cancer	135 (51.1)	30 (51.7)	105 (41.0)	
Esophageal cancer	73 (27.7)	15 (25.9)	58 (28.2)	
Others^a^	56 (21.2)	13 (22.4)	43 (20.9)	
**PD-1 inhibitor (n, %)**				0.331
Pembrolizumab	68 (25.8)	16 (27.6)	52 (25.2)	
Nivolumab	32 (12.1)	6 (10.3)	26 (12.6)	
Sintilimab	62 (23.5)	18 (31.0)	44 (21.4)	
Camrelizumab	8 (3.0)	2 (3.4)	6 (2.9)	
Toripalimab	35 (13.3)	3 (5.2)	32 (15.5)	
Tislelizumab	37 (14.0)	8 (13.8)	29 (14.1)	
Serplulimab	14 (5.3)	2 (3.4)	12 (5.8)	
Others^b^	8 (3.0)	3 (5.2)	5 (2.4)	
**Treatment phase of PD-1 inhibitor (n, %)**				0.190
Neoadjuvant or postoperative adjuvant	27 (10.2)	4 (6.9)	23 (11.2)	
First line	228 (86.4)	54 (93.1)	174 (84.5)	
Second and later line	9 (3.4)	0 (0.0)	9 (4.4)	
**Therapy (n, %)**				0.514
PD-1 inhibitor alone	8 (3.0)	1 (1.7)	7 (3.4)	
Combined with chemotherapy	181 (68.6)	40 (69.0)	141 (68.4)	
Combined with targeted drug	17 (6.4)	6 (10.3)	11 (5.3)	
Combined with chemotherapy and targeted drug	58 (22.0)	11 (19.0)	47 (22.8)	
**Pre-treatment**				
NLR (median, IQR)	2.6 (2.0,4.0)	2.6 (2.0,3.8)	2.6 (2.0,4.0)	0.717
PLR (median, IQR)	153.8 (112.5,220.8)	152.7 (105.6,221.0)	154.7 (114.7,220.4)	0.633
TSH (median, IQR)	1.82 (1.17,2.56)	1.6 (1.1,2.6)	1.9 (1.2,2.5)	0.411
TgAb (n, %)				0.372
Positive	28 (10.6)	8 (13.8)	20 (9.7)	
Negative	236 (89.4)	50 (86.2)	186 (90.3)	
TPOAb (n, %)				0.001
Positive	21 (8.0)	11 (19.0)	10 (4.9)	
Negative	243 (92.0)	47 (81.0)	196 (95.1)	
**Early stage of treatment**				
NLR (median, IQR)	2.4 (1.7,3.3)	2.3 (1.7,2.7)	2.4 (1.7,3.5)	0.249
PLR (median, IQR)	137.6 (103.2,190.5)	132.9 (105.5,158.7)	140.4 (102.7,197.7)	0.364
TSH (median, IQR)	1.50 (1.02,2,12)	1.0 (0.2,1.6)	1.6 (1.1,2.1)	0.000
TgAb (n, %)				0.000
Positive	27 (10.2)	14 (24.1)	13 (6.3)	
Negative	237 (89.8)	44 (75.9)	193 (93.7)	
TPOAb (n, %)				0.000
Positive	23 (8.7)	17 (29.3)	6 (2.9)	
Negative	241 (91.3)	41 (70.7)	200 (97.1)	

^a^including gastric cancer, liver cancer, bladder cancer, etc, ^b^including zimberelimab, penpulimab, etc, NLR, neutrophil-to-lymphocyte ratio; PLR, platelet-to-lymphocyte ratio; TSH, thyroid stimulating hormone; TgAb, anti-thyroglobulin antibody; TPOAb, anti-thyroperoxidase antibody.

### Biomarkers in the early treatment stage were superior for predicting irTD compared with pre-treatment markers

Considering the clinical significance, factors with *P* < 0.2 in the univariate logistic analysis were included in the multivariate logistic regression, clinical characteristics and pre-treatment hematological indicators were analyzed using logistic regression. Univariate analysis showed pre-treatment TPOAb was the potential factor. When subdividing irTD into thyrotoxicosis and hypothyroidism, thyrotoxicosis was correlated with gender, pre-treatment PLR, pre-treatment TSH and pre-treatment TgAb. The pre-treatment TSH level was the independent risk factor for thyrotoxicosis. Hypothyroidism was correlated with gender, BMI, pre-treatment TSH and pre-treatment TPOAb. Gender and pre-treatment TPOAb were the independent risk factors for hypothyroidism.

Logistic regression was performed on clinical characteristics and hematological indicators at the early stage of treatment. Univariate analysis showed that early-stage NLR, TgAb and TPOAb were correlated with irTD, and early-stage positive TgAb and TPOAb were independent risk factors for irTD. Thyrotoxicosis was correlated with gender, early-stage NLR, TSH level, TgAb and TPOAb, and early -stage TSH level was the independent risk factor of thyrotoxicosis. Gender, BMI, early-stage TSH level, TgAb and TPOAb were related to hypothyroidism, among which gender and early-stage TPOAb were the independent risk factors of hypothyroidism. The results of the multivariate logistic analysis were shown in **Table 2**, and the results of the univariate analysis were shown in **Supplementary Table S2**.

**Table 2:** Independent risk factors of irTD.

Independent risk factors	irTD		Thyrotoxicosis		Hypothyroidism	
	OR (95%CI)	*P* value	OR (95%CI)	*P* value	OR (95%CI)	*P* value
**Gender**	–	–	–	–	3.550 (1.376-9.163)	0.009
**Pre-treatment TSH**	–	–	0.636 (0.406-0.996)	0.048	–	–
**Pre-treatment TgAb**	–	–	–	–	–	–
**Pre-treatment TPOAb**	4.587 (1.840-11.437)	0.001	–	–	7.396 (2.540-21.530)	0.000
**Gender**	–	–	–	–	3.889 (1.457-10.380)	0.007
**Early-stage TSH**	–	–	0.162 (0.077-0.341)	0.000	–	–
**Early-stage TgAb**	2.831 (1.077-7.443)	0.035	–	–	–	–
**Early-stage TPOAb**	9.565 (3.399-26.921)	0.000	–	–	8.678 (2.656-28.357)	0.000

irTD, immune related thyroid dysfunction induced by PD-1 inhibitor; TSH, thyroid stimulating hormone; TgAb, anti-thyroglobulin antibody; TPOAb, anti-thyroperoxidase antibody.

**Figure 1:**
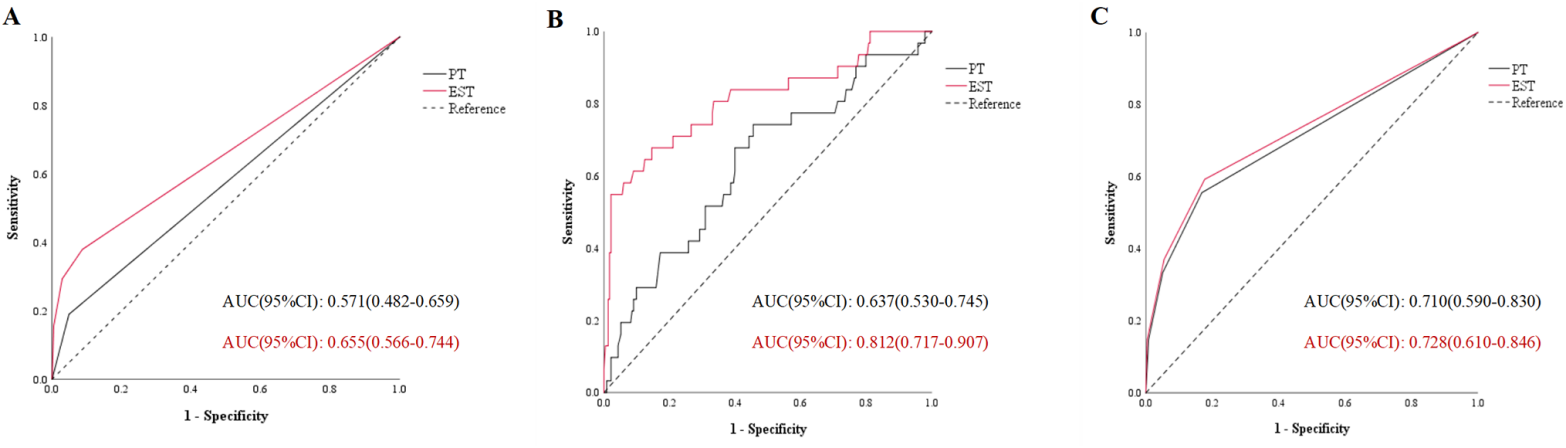
The area under the curve of immune related thyroid dysfunction **(A)**, thyrotoxicosis **(B)** and hypothyroidism **(C)**. PT, pre-treatment; EST, early stage of treatment; AUC, area under the curve.

ROC curves were utilized to assess the predictive capacity of pre-treatment biomarkers and biomarkers in early treatment stage for irTD. In the ROC curves of irTD, the AUC of indicators in early treatment stage was larger than that of pre-treatment indicators. This trend was consistent for both thyrotoxicosis and hypothyroidism, indicating that biomarkers at early treatment stage exhibited higher predictive accuracy. The ROC curves were shown in **Figure 1**.

### Predicting irTD with cytokines in the early treatment stage

**Figure 2:**
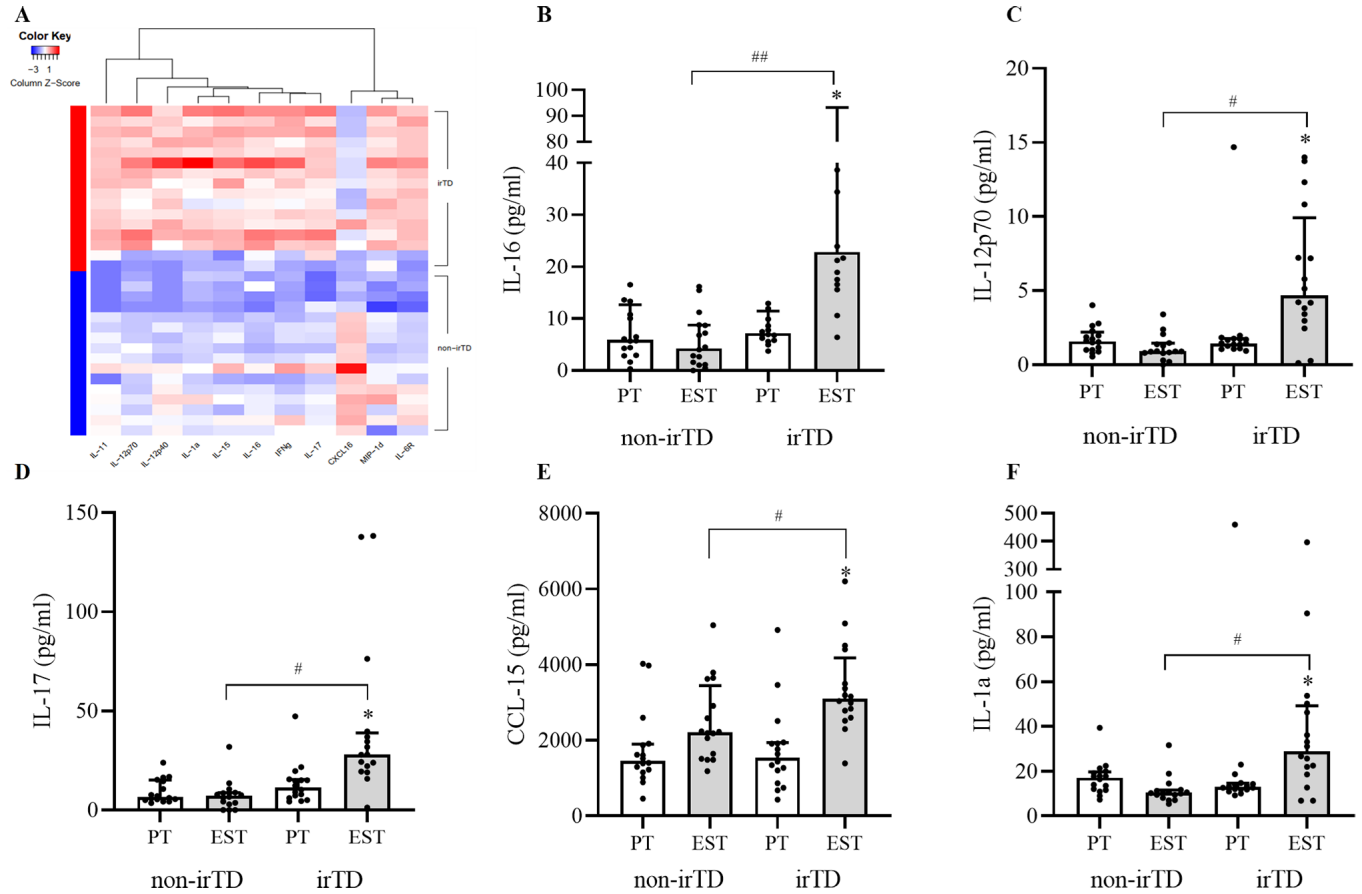
Differential proteins at the early stage of treatment **(A)**, differential proteins correlated with irTD **(B–F)** irTD: immune-related thyroid dysfunction induced by PD-1 inhibitor, PT, pre-treatment; EST, early stage of treatment, *significant difference between pre-treatment and early-stage cytokines in irTD group, *P*<0.05, ^#^significant difference between irTD and non-irTD group of cytokines at early stage, *P*<0.05, ^##^significant difference between irTD and non-irTD group of cytokines at early stage, *P*<0.01.

120 pre-treatment inflammatory and immune-related cytokines levels showed no differences between irTD and non-irTD group. There were significant differences of 11 cytokines at the early stage of treatment between the two groups. Compared with non-irTD group, IL-16, IL-11, IL-12p70, IL-17, CCL-15, IL-1a, IL-6R, IL-12p40, IL-15, IFNγ in irTD group were significantly increased and CXCL16 decreased significantly (**Figure 2A**).

Change of non-irTD group might also be a contributing factor for resulting in differential proteins. Differential proteins in the irTD group before and after treatment were also closely related to irTD. Based on this, our study further screened for differential proteins at early treatment phase between irTD and non-irTD group, with those same differential proteins in the irTD group before and after treatment, excluding the differential proteins in the non-irTD group before and after treatment. Ultimately, 5 cytokines closely correlated with irTD were identified, including IL-16, IL-12p70, IL-17, CCL-15 and IL-1a. All these cytokines were significantly increased in the irTD group compared with non-irTD group at the early stage of treatment [IL-16 (adjusted *P*=0.004), IL-12p70 (adjusted *P*=0.014), IL-17 (adjusted *P*=0.014), CCL-15 (adjusted *P*=0.014) and IL-1a (adjusted *P*=0.021)], and they were also increased significantly at the early stage of treatment compared with pre-treatment in the irTD group [IL-16 (adjusted *P*=0.010), IL-12p70 (adjusted *P*=0.010), IL-17 (adjusted *P*=0.035), CCL-15 (adjusted *P*=0.035) and IL-1a (adjusted *P*=0.018)] (**Figures 2B–F**) (**Supplementary Table S3**).

## Discussion

IrAEs can be triggered by immune checkpoint inhibitors (ICIs), impacting various organ systems including skin, lung, endocrine glands, digestive system and so on (13). IrAEs not only diminish the quality of life but also restrict the use of PD-1 inhibitor, and some severe cases can be life-threatening (14). Early identification and personalized intervention of irAEs are crucial for enhancing treatment effectiveness and safety, as well as managing healthcare costs. Research on predictive biomarkers play a vital role in early recognition and intervention of irAEs. Our study revealed that certain hematological markers at the early stage of treatment could predict irTD, and had stronger predictive capabilities compared to pre-treatment. Analyzing cytokine levels before and in the early stage of treatment in this study not only identified potential predictive cytokines for irTDs but also further underscored that biomarkers in the early stage of treatment have greater predictive value. To our knowledge, this was the first study to investigate the predictive potential of early-stage biomarkers for irTDs.

Previous studies have confirmed a significant association between post-treatment biomarkers and irAEs (6, 15). This association might be attributed to the unique immune response spectrum of irAEs patients to ICIs, rather than the patients’ immune characteristics. NG Nuñez, et al. showed that the expression of CXCL9, CXCL10, CXCL11 and IFN-g increased in irAEs patients within 1-2 weeks after ICIs treatment, along with significantly expansion of Ki-67+ regulatory T cells (Tregs) and Ki-67+ CD8+ T cells (16). R Reschke, et al. showed that activated CD4+ and CD8+ T cells, as well as CD4+/CD8+ effector memory T cells, increased before the second cycle of ICI treatment in irAEs patients (17). The fold change of autoantibody concentration from baseline to 6 weeks was related to specific irAEs (18). Early-stage biomarkers not only reflected patients’ characteristics but also screened high-risk patients with immune responses related to irAEs. These unique immune response characteristics required further clarification.

Research has shown that autoantibodies were closely associated with irTD (19). The chi-square test revealed that no significant difference was seen in the titers of pre-treatment TgAb between the irTD and non-irTD group. However, after treatment, higher positive TgAb was observed in irTD group compared with non-irTD group. Changes in autoantibody titers might be related to the B cells early changes induced by ICIs treatment (20). On one hand, ICIs treatment enhanced B cells activation and function, leading to the production of antibodies against non-tumor cell antigens, increasing the risk of autoimmune reactions. On the other hand, ICIs indirectly regulated B cells by modulating T cells, thereby triggering the production of autoantibodies (21).

This study delved into the predictive biomarkers for irTD, with a distinct focus on both thyrotoxicosis and hypothyroidism. The predictive biomarkers for thyrotoxicosis and hypothyroidism were found to be consistent between pre-treatment and post-treatment, with the ROC curve analysis revealing a significantly stronger predictive power in the early stage of treatment than pre-treatment. The limited sample size during subgroup analysis, might have contributed to the lack of additional indicators identified in the early treatment stage, highlighting the need for further exploration with larger sample size.

The levels of serum cytokines, which reflect immune status, are considered potential predictive biomarkers for irAEs (10). Investigating cytokine signatures associated with irTD is crucial due to the possibility of organ-specific irAEs having unique cytokine profiles (22). Existing researches on cytokine prediction of irTD were rather limited, with one study examining only 18 cytokines for irTD, and amidst patients had heterogeneous baseline characteristics (23). Our study assessed 120 cytokines, including inflammatory and immune related cytokines, while controlling for confounding factors such as sex, age, and tumor type. No pre-treatment cytokines were identified that could predict irTD. We observed 11 differential cytokines at the early stage of treatment between irTD and non-irTD group. Considering the possibility that these 11 differential cytokines might be attributed to fluctuations in non-irTD group, and the differential proteins observed in irTD group before and after treatment were related to the development of irTD. By scrutinizing differential proteins between the two groups in the early treatment stage and the differential proteins in irTD group between pre- and post-treatment, and subsequently excluding the differential proteins of the non-irTD group between pre- and post-treatment, IL-16, IL-12p70, IL-17, CCL-15 and IL-1a were identified as predictive cytokines of irTD. The above discussion of cytokine levels reconfirmed that patients were in a specific immune response state after receiving ICIs, and the relevant biomarkers at early stage were more valuable for predicting irTDs. The upregulation of IL-12p70, IL-17 and IL-1a in the irTD group aligned with previous studies on cytokine level associated with irAEs (24, 25). Furthermore, a Mendelian randomization analysis had indicated the correlation between elevated serum level of IL-12p70 and an increased risk of Hashimoto’s thyroiditis (26). IL-16has been found to be elevated in patients with autoimmune thyroid diseases such as Hashimoto’s thyroiditis, and has shown correlation with FT3, FT4 and TRAb levels (27). CCL-15has demonstrated increased expression in autoimmune diseases such as rheumatoid arthritis and asthma (28). It has indicated that these 5 cytokines were associated with irAEs or autoimmune diseases, especially autoimmune thyroiditis.

CD4+ T cells were shown to be essential for the occurrence of irTD (29). CD4+ T cells can differentiate into various functional subsets, such as Th1 and Th17. Several studies confirmed irAEs performing with Th1-skewed phenotype, as well as Th1/Th17 cell signatures (30, 31). Besides enhanced Th17, previously study has also shown that inhibition of IL-17A secreted by Th17, significantly reduced irTD development in ICI-treated mice, which indicated the pivotal role of IL-17 axis in irTD (32). The cytokine biomarkers IL-16, IL-1, IL-12 and IL-17, were all associated with CD4+ T cells. Among them, IL-16 has the function of recruiting CD4+ T cells to the site of inflammation and promoting their proliferation and differentiation (33). IL-12 and IL-1 respectively promote the differentiation of Th1 and Th17, while IL-17, secreted by Th17, mediates inflammation and is a key active molecule (34, 35). This might be the potential mechanism by which cytokine biomarkers were involved in the pathogenesis of irTD. Although CCL-15 has not been clearly associated with CD4+ T cells, protein-protein interaction (PPI) analysis using the STRING platform has shown that there was interaction of CCL-15 with IL-1 and IL-16 (**Supplementary Material S2**). Further research was needed to confirm the pathogenic mechanism of irTD.

This study had limitations as a single-center retrospective analysis with potential confounding factors and a limited sample size in the irTD group. Future studies with larger sample sizes were required to validate the conclusions, especially during subgroup analysis. There was currently no consensus of the optimal time for detecting predictive biomarkers in the early stage of treatment, and the application of cytokines as predictive biomarkers also required further in-depth research.

## Conclusion

In conclusion, biomarkers at the early treatment stage could identify individuals at high risk of developing irTD. These markers in the early treatment stage possessed a stronger predictive power compared with pre-treatment markers. Increased IL-16, IL-12p70, IL-17, CCL-15 and IL-1a in the early treatment stage had potential predictive ability of irTD.

## Data Availability

The original contributions presented in the study are included in the manuscript/**Supplementary Material**, further inquiries can be directed to the corresponding author.
