# Supplementary figures and images for: Biomarkers in the early stage of PD-1 inhibitor treatment have shown superior predictive capabilities for immune-related thyroid dysfunction
Source: Front Immunol. 2024 Oct 10;15:1458488. doi: 10.3389/fimmu.2024.1458488 (PMC11499093; doi:10.3389/fimmu.2024.1458488)

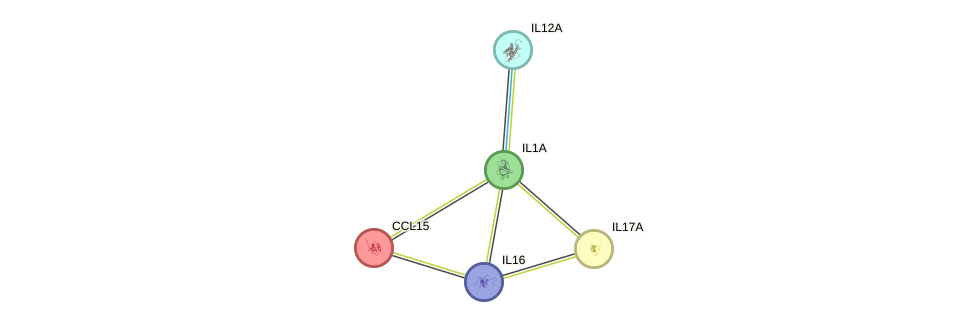

Supplement: Supplementary file 1 [file Image1.png]

**Supplementary material 1. The list of the investigated cytokines**


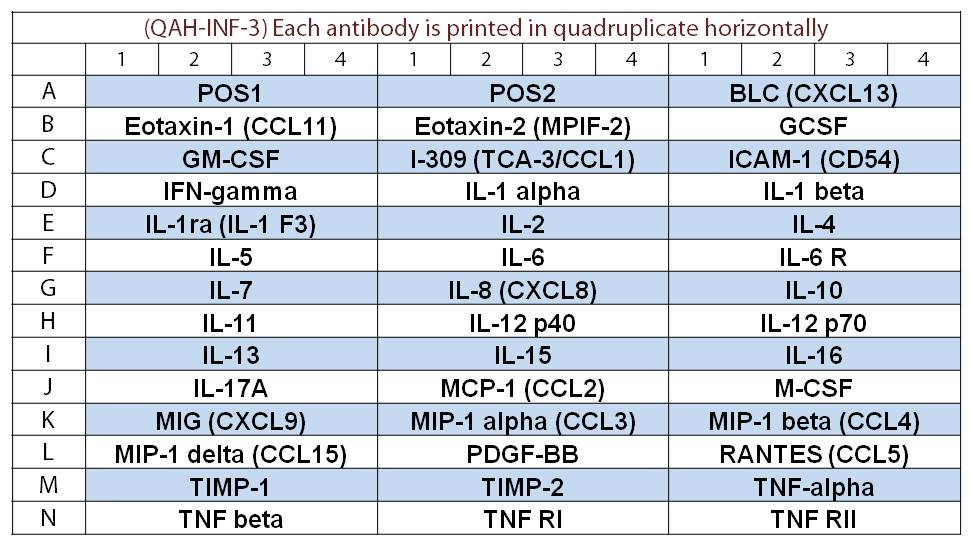


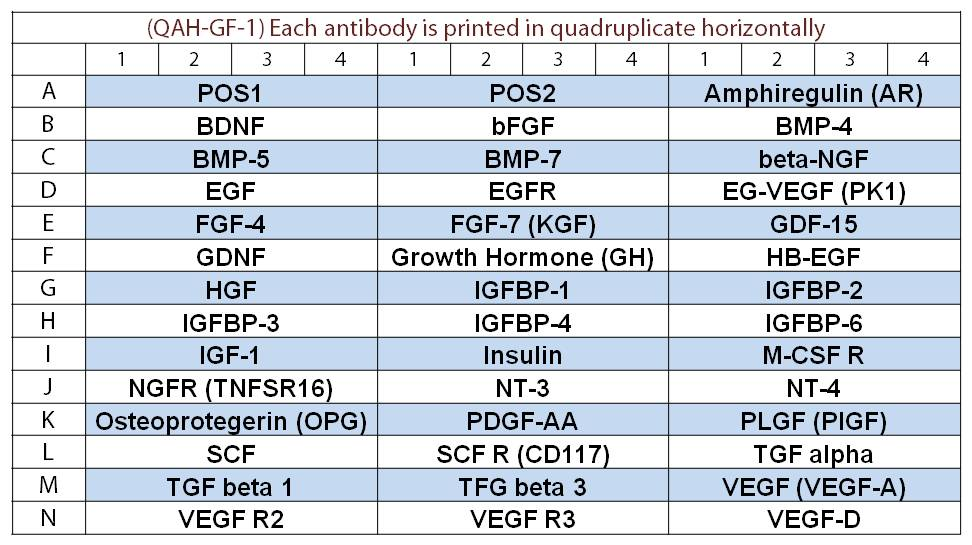


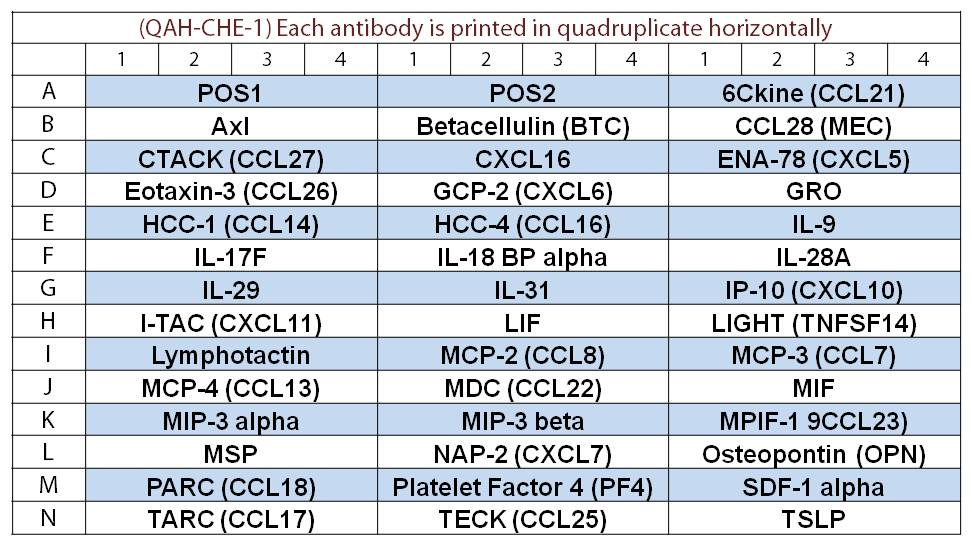

Supplement: Supplementary file 2 [file Table1.docx]
