# Supplementary material for: Biomarkers in the early stage of PD-1 inhibitor treatment have shown superior predictive capabilities for immune-related thyroid dysfunction
Source: Front Immunol. 2024 Oct 10;15:1458488. doi: 10.3389/fimmu.2024.1458488 (PMC11499093; doi:10.3389/fimmu.2024.1458488)
Supplement: Supplementary file 3 [file Table2.docx]

**Supplementary table1. The summary of irAEs in patients treated with PD-1 inhibitor**

| **Patients** | **Cancer** | **PD-1 inhibitor** | **irAEs** | **Grade** | **Time to onset (days)** | **irAE treatment** | **irAE caused discontinuation of PD-1 inhibitor** | **Rechallenge with PD-1 inhibitors** |
| --- | --- | --- | --- | --- | --- | --- | --- | --- |
| 1 | Gastric | Cadonilimab | Subclinical thyrotoxicosis | 1 | 54 | Observation | No |  |
| 2 | Bladder | Penpulimab | Overt hypothyroidism | 2 | 52 | Close monitoring | No |  |
| 3 | Pancreatic | Toripalimab | Myocarditis | 2 | 28 | Symptomatic treatment+Steroids+immunosuppression | Yes | Not known |
| 4 | Lung | Serplulimab | Overt hypothyroidism | 2 | 55 | Close monitoring | No |  |
| 5 | Lung | Serplulimab | Overt thyrotoxicosis | 2 | 53 | Close monitoring | No |  |
| 6 | Kidney | Nivolumab | Hepatitis | 3 | 67 | Symptomatic treatment+Steroids | No |  |
| 7 | Lung | Nivolumab | Overt hypothyroidism | 2 | 20 | Symptomatic treatment | No |  |
| 8 | Lung | Nivolumab | Overt hypothyroidism | 2 | 75 | Close monitoring | No |  |
| 9 | Kidney | Nivolumab | Myocarditis | 2 | 44 | Symptomatic treatment | Yes | Yes |
|  |  |  | Subclinical thyrotoxicosis | 1 | 147 | Observation |  |  |
| 10 | Esophageal | Nivolumab | Overt hypothyroidism | 2 | 74 | Close monitoring | No |  |
| 11 | Esophageal | Nivolumab | Myocarditis | 1 | 35 | Steroids | Yes | Yes |
| 12 | Esophageal | Tislelizumab | Subclinical thyrotoxicosis | 1 | 91 | Observation | No |  |
| 13 | Esophageal | Tislelizumab | Subclinical thyrotoxicosis | 1 | 17 | Observation | No |  |
| 14 | Lung | Sintilimab | Overt hypothyroidism | 2 | 21 | Close monitoring | No |  |
| 15 | Esophageal | Sintilimab | Rash | 3 | 60 | Symptomatic treatment | Yes | Not known |
| 16 | colorectal | Sintilimab | Overt hypothyroidism | 2 | 27 | Symptomatic treatment | No |  |
| 17 | Lung | Sintilimab | Overt thyrotoxicosis | 2 | 57 | Close monitoring | No |  |
| 18 | Lung | Sintilimab | Overt hypothyroidism | 2 | 136 | Close monitoring | No |  |
| 19 | Kidney | Sintilimab | Overt thyrotoxicosis | 2 | 25 | Close monitoring | No |  |
| 20 | Gastric | Sintilimab | Subclinical hypothyroidism | 1 | 126 | Observation | No |  |
| 21 | Kidney | Pembrolizumab | Subclinical thyrotoxicosis | 1 | 51 | Observation | No |  |
| 22 | Esophageal | Toripalimab | Overt thyrotoxicosis | 2 | 60 | Close monitoring | No |  |
| 23 | Lung | Tislelizumab | Subclinical thyrotoxicosis | 1 | 26 | Observation | No |  |
| 24 | Esophageal | Pembrolizumab | Subclinical hypothyroidism | 1 | 133 | Observation | No |  |
| 25 | Lung | Sintilimab | Overt thyrotoxicosis | 2 | 44 | Close monitoring | No |  |
| 26 | Esophageal | Sintilimab | Subclinical thyrotoxicosis | 1 | 59 | Observation | No |  |
| 27 | Lung | Tislelizumab | Overt thyrotoxicosis | 2 | 44 | Close monitoring | No |  |
| 28 | Bladder | Toripalimab | Overt hypothyroidism | 2 | 29 | Close monitoring | No |  |
| 29 | Lung | Pembrolizumab | Subclinical thyrotoxicosis | 1 | 51 | Observation | No |  |
| 30 | urothelial | Penpulimab | Subclinical thyrotoxicosis | 1 | 26 | Observation | No |  |
| 31 | Esophageal | Toripalimab | Subclinical thyrotoxicosis | 1 | 50 | Observation | No |  |
| 32 | Lung | Camrelizumab | Subclinical hypothyroidism | 1 | 70 | Observation | No |  |
| 33 | Gastric | Sintilimab | Overt hypothyroidism | 2 | 92 | Close monitoring | No |  |
| 34 | Esophageal | Pembrolizumab | Overt thyrotoxicosis | 2 | 25 | Symptomatic treatment | No |  |
| 35 | Liver | Pembrolizumab | Subclinical hypothyroidism | 1 | 121 | Observation | No |  |
| 36 | Gastric | Sintilimab | Overt hypothyroidism | 2 | 65 | Close monitoring | No |  |
| 37 | ovarian | Pembrolizumab | Subclinical hypothyroidism | 1 | 23 | Observation | No |  |
| 38 | Lung | Pembrolizumab | Subclinical thyrotoxicosis | 1 | 82 | Observation | No |  |
| 39 | Esophageal | Tislelizumab | Subclinical thyrotoxicosis | 1 | 80 | Observation | No |  |
| 40 | Esophageal | Camrelizumab | Subclinical thyrotoxicosis | 1 | 99 | Observation | No |  |
| 41 | Gastric | Sintilimab | Overt hypothyroidism | 2 | 130 | Close monitoring | No |  |
| 42 | Esophageal | Pembrolizumab | Subclinical thyrotoxicosis | 1 | 78 | Observation | No |  |
| 43 | Esophageal | Pembrolizumab | Overt hypothyroidism | 2 | 55 | Close monitoring | No |  |
| 44 | Lung | Sintilimab | Subclinical hypothyroidism | 1 | 61 | Observation | No |  |
| 45 | Gastric | Sintilimab | Overt thyrotoxicosis | 2 | 86 | Close monitoring | No |  |
| 46 | Esophageal | Pembrolizumab | Enteritis | 2 | 30 | Symptomatic treatment | No |  |
|  |  |  | Subclinical thyrotoxicosis | 1 | 87 | Observation |  |  |
| 47 | Lung | Sintilimab | Subclinical thyrotoxicosis | 1 | 21 | Observation | No |  |
| 48 | Lung | Nivolumab | Subclinical thyrotoxicosis | 1 | 219 | Observation | No |  |
| 49 | Lung | Nivolumab | Subclinical thyrotoxicosis | 1 | 472 | Observation | No |  |
| 50 | Lung | Nivolumab | Pneumonitis | 1 | 128 | Steroids | Yes | No |
| 51 | Lung | Pembrolizumab | Overt hypothyroidism | 2 | 93 | Close monitoring | No |  |
| 52 | Lung | Pembrolizumab | Overt hypothyroidism | 2 | 359 | Symptomatic treatment | No |  |
| 53 | Lung | Pembrolizumab | Pneumonitis | 2 | 43 | Symptomatic treatment+Steroids | Yes | No |
| 54 | Lung | Pembrolizumab | Overt thyrotoxicosis | 2 | 52 | Symptomatic treatment | No |  |
| 55 | Lung | Pembrolizumab | Pneumonitis | 2 | 96 | Symptomatic treatment | Yes | Yes |
| 56 | Lung | Pembrolizumab | Subclinical thyrotoxicosis | 1 | 73 | Observation | No |  |
| 57 | Lung | Pembrolizumab | Subclinical hypothyroidism | 1 | 174 | Observation | No |  |
| 58 | Lung | Pembrolizumab | Subclinical hypothyroidism | 1 | 401 | Observation | No |  |
| 59 | Lung | Pembrolizumab | Pneumonitis | 2 | 21 | Symptomatic treatment+Steroids | Yes | Not known |
| 60 | Lung | Pembrolizumab | Hypophysitis | 1 | 40 | Close monitoring | Yes | Not known |
| 61 | Lung | Sintilimab | Subclinical hypothyroidism | 1 | 65 | Symptomatic treatment | No |  |
| 62 | Lung | Sintilimab | Overt thyrotoxicosis | 2 | 32 | Close monitoring | No |  |
| 63 | Lung | Sintilimab | Overt hypothyroidism | 2 | 120 | Close monitoring | No |  |
| 64 | Lung | Sintilimab | Rash | 3 | 23 | Symptomatic treatment+Steroids | Yes | No |
|  |  |  | Subclinical thyrotoxicosis | 1 | 49 | Observation |  |  |
| 65 | Lung | Tislelizumab | Pneumonitis | 1 | 163 | Symptomatic treatment | Yes | No |
| 66 | Lung | Tislelizumab | Overt hypothyroidism | 2 | 22 | Close monitoring | No |  |
| 67 | Lung | Tislelizumab | Myocarditis | 2 | 46 | Steroids | Yes | No |
| 68 | Lung | Tislelizumab | Pneumonitis | 2 | 329 | Symptomatic treatment+Steroids | Yes | No |
|  |  |  | Subclinical thyrotoxicosis | 1 | 345 | Observation |  |  |
| 69 | Lung | Tislelizumab | Pneumonitis | 1 | 416 | Symptomatic treatment+Steroids | No |  |
|  |  |  | Overt hypothyroidism | 2 | 489 | Close monitoring |  |  |
| 70 | Lung | Toripalimab | Kidney Injury | 2 | 33 | Symptomatic treatment | Yes | Yes |
|  |  |  | Diabetes | 2 | 345 | Symptomatic treatment |  |  |

irAEs: Immune-related adverse events
