# Supplementary material for: Biomarkers in the early stage of PD-1 inhibitor treatment have shown superior predictive capabilities for immune-related thyroid dysfunction
Source: Front Immunol. 2024 Oct 10;15:1458488. doi: 10.3389/fimmu.2024.1458488 (PMC11499093; doi:10.3389/fimmu.2024.1458488)
Supplement: Supplementary file 4 [file Table3.docx]

**Supplementary Table 2 Univariate logistic regression of risk factors at pre-treatment and at early stage of treatment for irTD**

|  | **irTD**  **OR(95%CI)** | ***P* value** | **Thyrotoxicosis OR(95%CI)** | ***P* value** | **Hypothyroidism OR(95%CI)** | ***P* value** |
| --- | --- | --- | --- | --- | --- | --- |
| **Gener** | 1.428(0.665-3.070) | 0.361 | 0.166(0.022-1.252) | 0.082 | 4.059(1.701-9.687) | 0.002 |
| **Age** | 0.996(0.968-1.025) | 0.801 | 0.999(0.962-1.036) | 0.938 | 0.995(0.957-1.034) | 0.794 |
| **BMI** | 0.945(0.863-1.034) | 0.219 | 0.983(0.877-1.102) | 0.774 | 0.916(0.807-1.038) | 0.170 |
| **History of thyroid disease** | 1.198(0.371-3.863) | 0.763 | 0.484(0.062-3.801) | 0.490 | 2.154(0.573-8.096) | 0.256 |
| **Pre-treatment NLR** | 0.986(0.906-1.072) | 0.735 | 0.922(0.782-1.087) | 0.333 | 1.023(0.940-1.113) | 0.603 |
| **Pre-treatment PLR** | 0.999(0.997-1.002) | 0.534 | 0.994(0.989-1.000) | 0.042 | 1.000(0.999-1.001) | 0.730 |
| **Pre-treatment TSH** | 0.962(0.731-1.267) | 0.783 | 0.629(0.407-0.972) | 0.037 | 1.378(0.982-1.932) | 0.063 |
| **Pre-treatment TgAb** | 1.488(0.619-3.578) | 0.375 | 2.302(0.852-6.215) | 0.100 | 0.649(0.145-2.900) | 0.572 |
| **Pre-treatment TPOAb** | 4.587(1.840-11.437) | 0.001 | 0.777(0.172-3.508) | 0.743 | 9.375(3.488-25.196) | 0.000 |
| **NLR** **at early stage of treatment** | 0.815(0.650-1.022) | 0.076 | 0.812(0.601-1.098) | 0.177 | 0.855(0.632-1.157) | 0.310 |
| **PLR at early stage of treatment** | 0.999(0.996-1.002) | 0.541 | 0.997(0.991-1.002) | 0.214 | 1.001(0.997-1.005) | 0.635 |
| **TSH at early stage of treatment** | 1.020(0.943-1.103) | 0.628 | 0.154(0.077-0.311) | 0.000 | 1.115(0.945-1.317) | 0.198 |
| **TgAb at early stage of treatment** | 4.724(2.075-10.756) | 0.000 | 2.423(0.894-6.569) | 0.082 | 4.831(1.869-12.489) | 0.001 |
| **TPOAb at early stage of treatment** | 13.821(5.138-37.177) | 0.000 | 3.956(1.480-10.573) | 0.006 | 10.136(3.879-26.487) | 0.000 |

irTD：immune related thyroid dysfunction by PD-1 inhibitor, NLR: neutrophil-to-lymphocyte ratio, PLR: platelet-to-lymphocyte ratio, TSH: thyroid stimulating hormone, TgAb: anti-thyroglobulin antibody, TPOAb: anti-thyroperoxidase antibody
